# Multiple endocrine neoplasia type 1 (MEN1) and type 4 (MEN4)

**DOI:** 10.1016/j.mce.2013.08.002

**Published:** 2014-04-05

**Authors:** Rajesh V. Thakker

**Affiliations:** Academic Endocrine Unit, Radcliffe Department of Medicine, University of Oxford, Oxford Centre for Diabetes, Endocrinology and Metabolism (OCDEM), Churchill Hospital, Headington, Oxford OX3 7LJ, United Kingdom

**Keywords:** Parathyroid, Pancreatic islet, Pituitary, Tumors, Neuroendocrine tumors, Histone methylation

## Abstract

Multiple endocrine neoplasia (MEN) is characterized by the occurrence of tumors involving two or more endocrine glands within a single patient. Four major forms of MEN, which are autosomal dominant disorders, are recognized and referred to as: MEN type 1 (MEN1), due to menin mutations; MEN2 (previously MEN2A) due to mutations of a tyrosine kinase receptor encoded by the rearranged during transfection (RET) protoncogene; MEN3 (previously MEN2B) due to RET mutations; and MEN4 due to cyclin-dependent kinase inhibitor (CDNK1B) mutations. Each MEN type is associated with the occurrence of specific tumors. Thus, MEN1 is characterized by the occurrence of parathyroid, pancreatic islet and anterior pituitary tumors; MEN2 is characterized by the occurrence of medullary thyroid carcinoma (MTC) in association with phaeochromocytoma and parathyroid tumors; MEN3 is characterized by the occurrence of MTC and phaeochromocytoma in association with a marfanoid habitus, mucosal neuromas, medullated corneal fibers and intestinal autonomic ganglion dysfunction, leading to megacolon; and MEN4, which is also referred to as MENX, is characterized by the occurrence of parathyroid and anterior pituitary tumors in possible association with tumors of the adrenals, kidneys, and reproductive organs. This review will focus on the clinical and molecular details of the MEN1 and MEN4 syndromes. The gene causing MEN1 is located on chromosome 11q13, and encodes a 610 amino-acid protein, menin, which has functions in cell division, genome stability, and transcription regulation. Menin, which acts as scaffold protein, may increase or decrease gene expression by epigenetic regulation of gene expression via histone methylation. Thus, menin by forming a subunit of the mixed lineage leukemia (MLL) complexes that trimethylate histone H3 at lysine 4 (H3K4), facilitates activation of transcriptional activity in target genes such as cyclin-dependent kinase (CDK) inhibitors; and by interacting with the suppressor of variegation 3–9 homolog family protein (SUV39H1) to mediate H3K methylation, thereby silencing transcriptional activity of target genes. MEN1-associated tumors harbor germline and somatic mutations, consistent with Knudson’s two-hit hypothesis. Genetic diagnosis to identify individuals with germline *MEN1* mutations has facilitated appropriate targeting of clinical, biochemical and radiological screening for this high risk group of patients for whom earlier implementation of treatments can then be considered. MEN4 is caused by heterozygous mutations of CDNK1B which encodes the 196 amino-acid CDK1 p27Kip1, which is activated by H3K4 methylation.

## 1 Introduction

Multiple endocrine neoplasia (MEN) is characterized by the occurrence of tumors involving two or more endocrine glands within a single patient (Thakker, 2010). The disorder has previously been referred to as multiple endocrine adenopathy or the pluriglandular syndrome. However, glandular hyperplasia and malignancy may also occur in some patients, and the term multiple endocrine neoplasia is now preferred. Four major forms of MEN are recognized and referred to as types 1–4 (MEN1–MEN4), and each form is characterized by the development of tumors within specific endocrine glands (Table 1) (Thakker, 1998). All these forms of MEN either may be inherited as autosomal-dominant syndromes (Thakker, 1998) or may occur sporadically; that is, without a family history (Thakker, 2010). However, this distinction between sporadic and familial cases may sometimes be difficult, because in some sporadic cases, a family history may be absent because the parent with the disease may have died before symptoms developed. MEN1, which is also referred to as Wermer’s syndrome (Thakker, 1998) is characterized by the combined occurrence of tumors of the parathyroid glands, the pancreatic islet cells, and the anterior pituitary. In addition to these tumors, adrenal cortical tumors, carcinoid, facial angiofibromas, collagenomas, and lipomatous tumors have been described in patients with MEN1 (Trump et al., 1996). However, in MEN2, which is also called Sipple’s syndrome and was previously referred to as MEN2A, medullary thyroid carcinoma (MTC) occurs in association with phaeochromocytoma and parathyroid tumors (Thakker, 1998). In MEN3, which was previously referred to as MEN2B, MTC and pheochromocytoma occur in association with a marfanoid habitus, mucosal neuromas, medullated corneal fibers, and intestinal autonomic ganglion dysfunction leading to megacolon (Thakker, 1998), with rare occurrence of parathyroid tumors. MTC may also occur as the sole manifestation of the MEN2 syndrome, and this variant is referred to as MTC-only. In MEN4, which is also referred to as MENX, patients develop parathyroid and anterior pituitary tumors, which may occur in association with tumors of the reproductive organs, adrenals and kidneys. This review will focus on the clinical features, genetics and epigenetics of the MEN1 and MEN4 syndromes.

## 2 Multiple endocrine neoplasia type 1 (MEN1)

### 2.1 Clinical features

The incidence of MEN1 has been estimated from randomly chosen postmortem studies to be 0.25% and to be 1–18% among patients with primary hyperparathyroidism, 16–38% among patients with gastrinomas, and less than 3% among patients with pituitary tumors (Thakker, 2010). The disorder affects all age groups, with a reported age range of 5–81 years, with clinical and biochemical manifestations of the disorder having developed in 80% and more than 98% of patients, respectively, by the fifth decade (Thakker et al., 1989, 2012; Trump et al., 1996). The clinical manifestations of MEN1 are related to the sites of tumors and to their products of secretion. Parathyroid tumors, resulting in primary hyperparathyroidism, are the most common feature of MEN1 and occur in ∼95% of MEN1 patients (Benson et al., 1987; Brandi et al., 2001; Calender et al., 1995; Marx et al., 1998; Trump et al., 1996). Pancreatic islet tumors, which are also referred to as pancreatic neuroendocrine tumors (NETs) consist of gastrinomas, insulinomas, pancreatic polypeptidomas (PPomas), glucagonomas, vaso-active intestinal polypeptidomas (VIPomas) and non-functioning tumors and these occur in ∼40% of MEN1 patients; and anterior pituitary tumors, consisting of prolactinomas, somatotrophinomas, corticotrophinomas, and non-functioning adenomas, occur in ∼30% of patients (Benson et al., 1987; Calender et al., 1995; Marx et al., 1998; Trump et al., 1996). In addition, some MEN1 patients may also develop adrenocortical tumors, lipomas, carcinoid tumors, facial angiofibromas and collagenomas (Bassett et al., 1998; Brandi et al., 2001; Calender et al., 1995; Marx et al., 1998; Trump et al., 1996). Parathyroid tumors are the first manifestation of MEN1 in more than 85% of patients, and in the remaining fewer than 15% of patients, the first manifestation may be an insulinoma or a prolactinoma (Thakker et al., 1989; Trump et al., 1996). Combinations of these affected glands and their pathologic features (for example, hyperplasia or single or multiple adenomas of the parathyroid glands) have been reported to differ in members of the same family (Thakker et al., 1989; Trump et al., 1996) and even between identical twins (Flanagan et al., 1996). MEN1 is inherited as an autosomal-dominant disorder in such families, but a non-familial (i.e., sporadic) (Trump et al., 1996) form may have developed in 8–14% of patients with MEN1, and molecular genetic studies have confirmed the occurrence of de novo mutations of the *MEN1* gene in approximately 10% of patients with MEN1 (Bassett et al., 1998). In the absence of treatment, these tumors have been observed to be associated with an earlier mortality in patients with MEN1 (Burgess et al., 1998). Studies of MEN1-related mortality have demonstrated that 28–46% of deaths are directly related to MEN1, most commonly as a result of malignant pancreatic islet tumors, gastrinomas and foregut carcinoids (Dean et al., 2000). In addition, these deaths occur at a significantly younger age than those occurring in unaffected individuals (Dean et al., 2000). Furthermore, a recent multicentre study from France and Belgium has suggested that ∼70% individuals with MEN1 currently die of causes directly related to MEN1 (Goudet et al., 2010). In particular, malignant pancreatic islet tumors and thymic carcinoid tumors were associated with a marked increased in risk of death (Hazard ratio >3, *P* < 0.005) (Goudet et al., 2010).

**Fig. 1:**
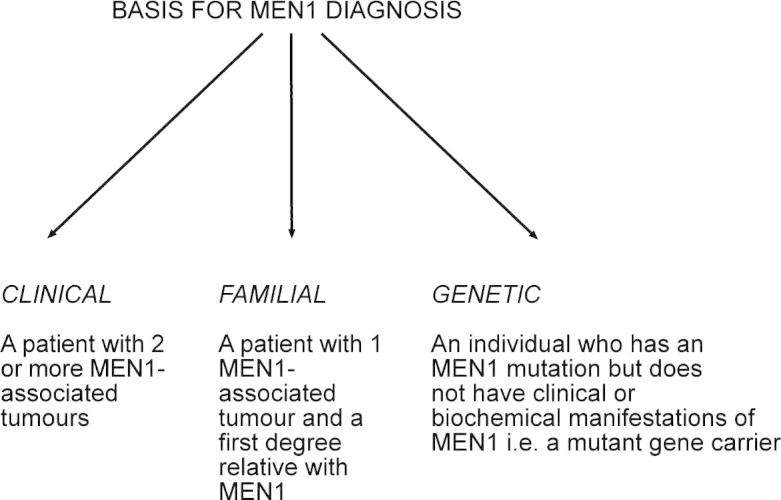
Basis for a diagnosis of MEN1 in individuals. A diagnosis of MEN1 based on clinical and familial criteria may be confounded by the occurrence of phenocopies. (Reproduced with permission from Turner et al. (2010).) (Turner et al., 2010).

**Fig. 2:**
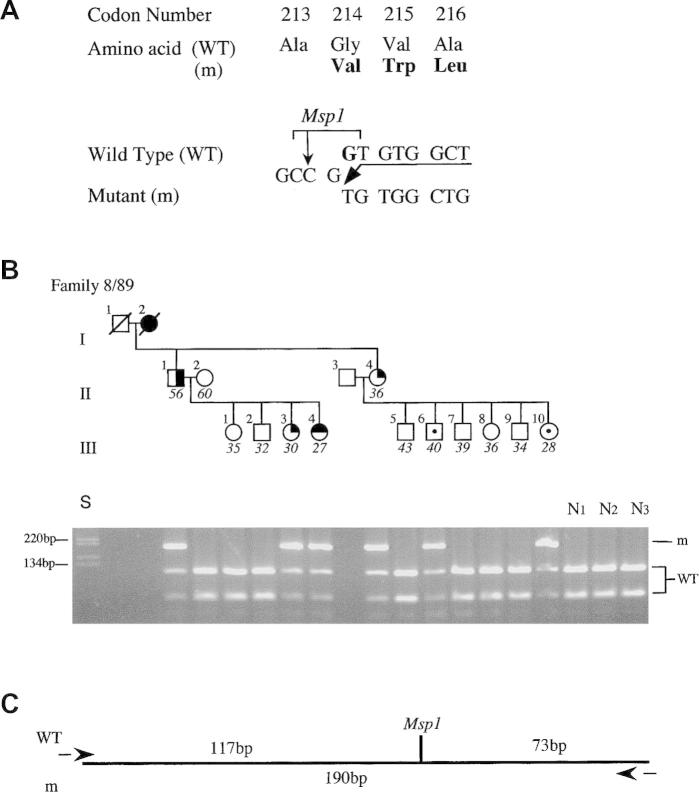
Detection of *MEN1* mutation in exon 3 in family 8/89 by restriction enzyme analysis. DNA sequence analysis of individual II.1 revealed a 1-bp deletion at the second position (GGT) of codon 214 (A). The deletion has caused a frameshift that continues to codon 223 before a stop codon (TGA) is encountered in the new frame. The 1-bp deletion results in the loss of an MspI restriction enzyme site (C/CGG) from the normal (wild-type, WT) sequence (A), and this finding has facilitated detection of this mutation in the other affected members (II.4, III.3, and III.4) of this family (B). The mutant (m) polymerase chain reaction product is 190 bp, whereas the WT products are 117 and 73 bp (C). The affected individuals were heterozygous, and the unaffected members were homozygous for the WT sequence. Individuals III.6 and III.10, who are 40 and 28 years old, respectively, are mutant gene carriers who are clinically and biochemically normal because of the age-related penetrance of this disorder (Fig. 5). These individuals would still require screening (Fig. 6) by clinical, biochemical, and radiologic assessment because they still have a residual risk (i.e., 100% – age-related penetrance) of 2% and >13%, respectively, of tumors developing by age 60 years. Individuals are represented as male (square); female (circle); unaffected (open); affected with parathyroid tumors (solid upper right quadrant), gastrinoma (solid lower right quadrant), or prolactinoma (solid upper left quadrant); and unaffected mutant gene carriers (dot in the middle of the open symbol). Individual I.2, who is dead but was known to be affected (tumor details not known), is shown as a solid symbol. The age is indicated below for each individual at diagnosis or at the time of the last biochemical screening. The standard size marker (S) in the form of the 1-kb ladder is indicated. Cosegregation of this mutation with MEN1 in family 8/89 and its absence in 110 alleles from 55 unrelated normal individuals (N1–3 shown) indicate that it is not a common DNA sequence polymorphism. (Adapted from Bassett JHD, Forbes SA, Pannett AAJ, et al.: Characterization of mutations in patients with multiple endocrine neoplasia type 1 (MEN1). *Am J Hum Genet* 62: 232–244, 1998, with permission.) (Bassett et al., 1998).

A diagnosis of MEN1 may be established in an individual by one of three criteria (Fig. 1). Thus, MEN1 may be clinically diagnosed in an individual on the basis of the occurrence of two or more MEN1-associated endocrine tumors (Fig. 1) (Thakker et al., 2012; Turner et al., 2010). In addition, a diagnosis of familial MEN1 is established for an individual who has the occurrence of one of the MEN1-associated tumors and is a first degree relative of a patient with a clinical diagnosis of MEN1. Finally, a genetic diagnosis of MEN1 is made on identification of a germline *MEN1* mutation in an individual, who may be asymptomatic and has not yet developed any of the serum biochemical or radiological abnormalities indicative of tumor development (Fig. 2) (Turner et al., 2010).

### 2.2 Treatment

The treatment for each type of MEN1 associated endocrine tumor is generally similar to that for the respective tumors occurring in non-MEN1 patients. However, the treatment outcomes of MEN1-associated tumors are not as successful as those in non-MEN1 patients, for several reasons. First, MEN1-associated tumors, with the exception of pituitary NETs, are usually multiple, thereby making it difficult to achieve a successful surgical cure. For example, MEN1 patients often develop multiple submucosal duodenal gastrinomas, thereby reducing surgical cure rates compared with similar sporadic solitary tumors, such that only ∼15% of MEN1 patients, compared to ∼45% of non-MEN1 patients, are free of disease immediately after surgery, and at 5 years this decreased to ∼5% in MEN1 patients, compared to ∼40% in non-MEN1 patients (Brandi et al., 2001; Imamura et al., 2011; Norton et al., 1999; Norton and Jensen, 2007; Tonelli et al., 2006). MEN1 patients also develop multiple parathyroid tumors, and subtotal parathyroidectomy has resulted in persistent or recurrent hypercalcaemia within 10 years in 20–60% of MEN1 patients, as opposed to ∼4% in non-MEN1 patients (Brandi et al., 2001; Schreinemakers et al., 2011; Waldmann et al., 2010). Secondly, occult metastatic disease is more prevalent in MEN1 patients with NETs than in patients with sporadic endocrine tumors. For example, metastases are present in up to 50% of patients with MEN1-associated insulinomas, whereas <10% of non-MEN1 insulinomas metastasise (Akerstrom and Hellman, 2007). Thirdly, MEN1-associated tumors may be larger, more aggressive, and resistant to treatment. For example, ∼85% of anterior pituitary tumors in MEN1 patients, as opposed to 64% in non-MEN1 patients are macroadenomas at the time of diagnosis; ∼30% of anterior pituitary tumors in MEN1 patients have invaded surrounding tissue (Hardy classification grades III and IV), compared to 10% in non-MEN1 patients; and >45% of anterior pituitary NETs in MEN1 patients had persistent hormonal over-secretion following appropriate medical, surgical and radiotherapy treatment, compared with between 10% and 40% in non-MEN1 patients (Fernandez et al., 2010; Trouillas et al., 2008; Verges et al., 2002). Thus, the treatment of MEN1-associated tumors is difficult requires a multi-disciplinary team of experts.

### 2.3 Genetics

**Fig. 3:**
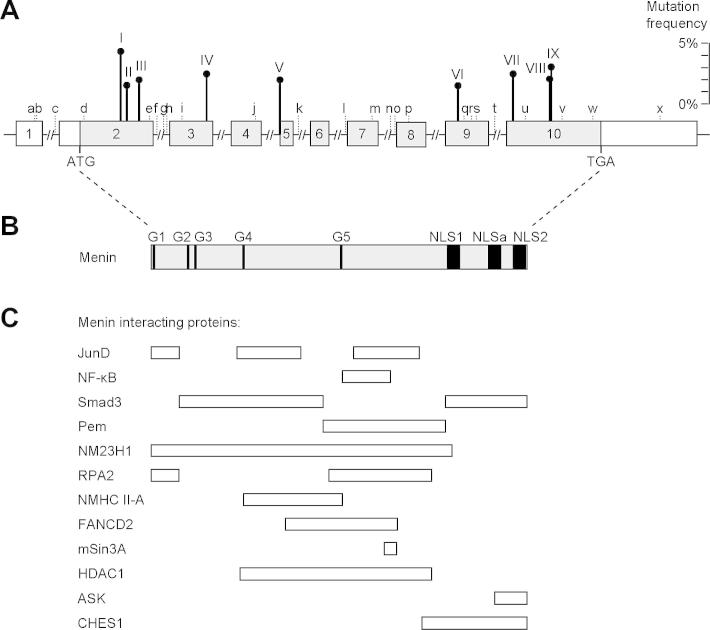
Schematic representation of the genomic organization of the *MEN1* gene, its encoded protein (menin) and regions that interact with other proteins. (A) The human *MEN1* gene consists of 10 exons that span more than 9 kb of genomic DNA and encodes a 610-amino acid protein. The 1.83 kb coding region (indicated by shaded region) is organized into 9 exons (exons 2–10) and 8 introns (indicated by a line but not to scale). The sizes of the exons (boxes) range from 41 to 1297 bp, and that of the introns range from 80 to 1564 bp. The start (ATG) and stop (TGA) codons in exons 2 and 10, respectively, are indicated. Exon 1, the 5′ part of exon 2, and the 3′ part of exon 10 are untranslated (indicated by open boxes). The promoter region is located within a few 100 bp upstream of exon 2. The sites of the nine germline mutations (I–IX) that occur with a frequency >1.5% (Table 2) are shown and their respective frequencies (scale shown on the right) are indicated by the vertical lines above the gene. These germline mutations, which collectively represent 20.6% of all reported germline mutations, are: I – c.249_252delGTCT; II – c.292C>T; III – c.358_360delAAG; IV4 – c.628_631delACAG; V – c.784−9G>A; VI – c.1243C>T; VII – c.1378C>T; VIII – c.1546delC; IX – c.1546_1547insC. The locations of the 24 polymorphisms (a–x, Table 4) are illustrated. (B) Menin has three nuclear localization signals (NLSs) at codons 479–497 (NLS1), 546–572 (NLSa) and 588–608 (NLS2), indicated by closed boxes, and five putative guanosine triphosphatase (GTPase) sites (G1–G5) indicated by closed bars. (C) Menin regions that have been implicated in the binding to different interacting proteins (Table 6) are indicated by open boxes. These are JunD (codons 1–40, 139–242, 323–428); nuclear factor-kappa B (NF-κB) (codons 305–381); Smad3 (codons 40–278, 477–610); placenta and embryonic expression, pem (codons 278–476); NM23H1 (codons 1–486); a subunit of replication protein A (RPA2) (codons 1–40, 286–448); NMHC II-A (codons 154–306); FANCD2 (codons 219–395); mSin3A (codons 371–387); HDAC1 (codons 145–450); ASK (codons 558–610) and CHES1 (codons 428–610). The regions of menin that interact with GFAP, vimentin, Smad 1/5, Runx2, MLL-histone methyltransferase complex and estrogen receptor-alpha remain to be determined. (Reproduced from Lemos MC, Thakker RV: Multiple Endocrine Neoplasia Type 1 (MEN1): Analysis of 1336 mutations reported in the first decade following identification of the gene. *Hum Mutat* 29: 22–32, 2008, with permission.) (Lemos and Thakker, 2008).

**Fig. 4:**
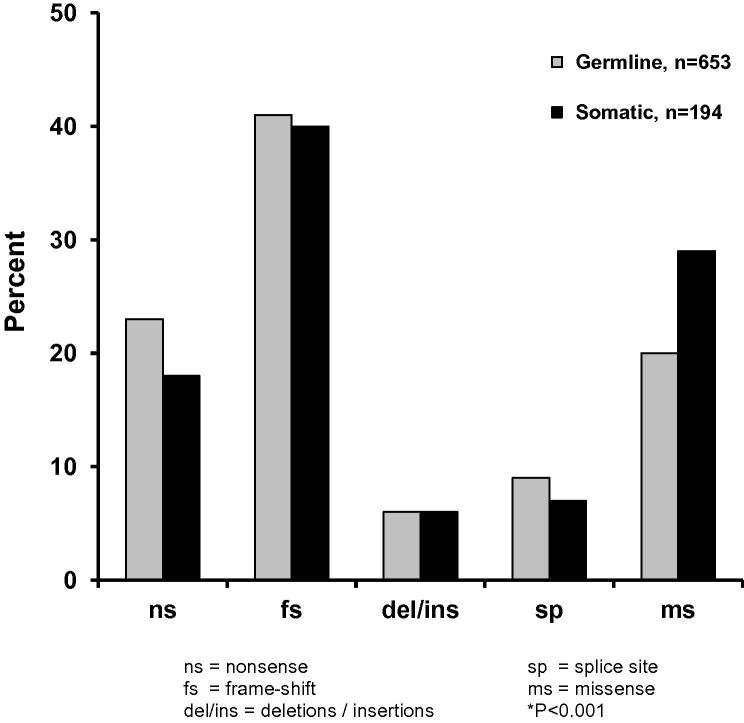
Frequency of germ-line and somatic *MEN1* mutations. A total of 1133 germ-line mutations and 203 somatic mutations have been reported (Agarwal et al., 1997), and these are of diverse types (e.g., nonsense, frameshifts, deletions, insertions, splice-site, and missense mutations). The frequencies of each type of mutation in the germ-line and somatic groups are similar, with the exception of the missense mutations, which are found more frequently in tumors (i.e., the somatic group). (Reproduced from Lemos MC, Thakker RV: Multiple Endocrine Neoplasia Type 1 (MEN1): Analysis of 1336 mutations reported in the first decade following identification of the gene. *Hum Mutat* 29: 22–32, 2008, with permission.) (Lemos and Thakker, 2008).

The *MEN1* gene is located on chromosome 11q13 and consists of 10 exons (Fig. 3) that encode a 610-amino acid protein, referred to as menin. Menin is ubiquitously expressed and is predominantly a nuclear protein in non-dividing cells. Mutations of the *MEN1* gene (Figs. 2 and 3) have been characterized, and a total of 1336 mutations (1133 germline and 203 somatic mutations) were reported in the first decade following the identification of the gene (Lemos and Thakker, 2008). The 1133 germ-line mutations of the *MEN1* gene, which consist of 459 different mutations are scattered throughout the entire 1.830 bp coding region and splice sites of the *MEN1* gene (Agarwal et al., 1997; Bassett et al., 1998; Chandrasekharappa et al., 1997; Lemos and Thakker, 2008; The European Consortium on MEN1, 1997). Approximately 23% are nonsense mutations, around 41% are frameshift deletions or insertions, 6% are in-frame deletions or insertions, 9% are splice-site mutations, and 20% are missense mutations, and 1% are whole or particular gene deletions (Fig. 4) (Lemos and Thakker, 2008). More than 10% of the *MEN1* mutations arise de novo and may be transmitted to subsequent generations (Bassett et al., 1998; Lemos and Thakker, 2008; Zablewska et al., 2003). It also is important to note that between 5% and 10% of patients with MEN1 may not harbor mutations in the coding region of the *MEN1* gene (Agarwal et al., 1997; Bassett et al., 1998; Chandrasekharappa et al., 1997; Lemos and Thakker, 2008; The European Consortium on MEN1, 1997), and that these individuals may have whole gene deletions or mutations in the promoter or untranslated regions, which remain to be investigated. One study showed that approximately 33% of patients who do not have mutations within the coding region have large deletions involving complete exons (Cavaco et al., 2002). Such abnormalities will not be easily detected by DNA sequence analysis.

Most (75%) of the *MEN1* mutations are inactivating (Lemos and Thakker, 2008) and are consistent with those expected in a tumor-suppressor gene. The mutations not only are diverse in their types but are also scattered throughout the 1830-bp coding region of the *MEN1* gene with no evidence for clustering as observed in MEN2 (Table 1). However, some of the mutations have been observed to occur several times in unrelated families (Fig. 3 and Table 2). Mutations at nine sites in the *MEN1* gene accounted for over 20% of all the germ-line mutations (Table 2). Of these nine types of mutations, five are deletional and insertional mutations involving codons 83 and 84 (nt359 del 4), 120 (Lys[K]120 del), 210–211 (nt 738 del 4), and codons 514–516 (nt 1656–7 del or ins C); one is a novel acceptor splice site in intron 4, and three are nonsense mutations (Arg98Stop, Arg415Stop, and Arg460Stop) (Lemos and Thakker, 2008). These mutations at these nine different sites could be considered to represent potential “hot” spots (Table 2). Such deletional and insertional hot spots may be associated with DNA sequence repeats, which may consist of long tracts of either single nucleotides or shorter elements ranging from dinucleotides to octanucleotides (Bassett et al., 1998). The DNA sequences in the vicinity of codons 83 and 84 in exon 2, and codons 210–211 in exon 3, contain CT and CA dinucleotide repeats, respectively, flanking the 4-bp deletions; this finding would be consistent with a replication-slippage model in which misalignment of the dinucleotide repeat takes place during replication, followed by excision of the 4-bp single-stranded loop (Bassett et al., 1998). A similar replication-slippage model may also be involved at codons 119 and 120, each of which consists of AAG nucleotides encoding a lysine (K) residue. The deletions and insertions of codon 516 involve a poly(C)_7_ tract, and a slipped-strand mispairing model also is the most likely mechanism to be associated with this mutational hot spot (Bassett et al., 1998; Lemos and Thakker, 2008). Thus, the *MEN1* gene appears to contain DNA sequences that may render it susceptible to deletional and insertional mutations.

Correlations between *MEN1* mutations and clinical manifestations of the disorder appear to be absent. For example, a detailed study of five unrelated families with the same 4-bp deletion in codons 210 and 211 (Table 3) revealed a wide range of MEN1-associated tumors (Bassett et al., 1998; Lemos and Thakker, 2008; Thakker, 1998); all affected family members had parathyroid tumors, but members of families 1, 3, 4, and 5 had gastrinomas, whereas members of family 2 had insulinomas. In addition, prolactinomas occurred in members of families 2, 3, 4, and 5 but not in family 1, which was affected with carcinoid tumors. Another study of seven unrelated families with the same g → a novel acceptor splice-site mutation in intron 4 revealed a similarly wide range of MEN1-associated tumors and a lack of genotype/phenotype correlation. The apparent lack of genotype/phenotype correlation, which contrasts with the situation in MEN2 (Table 1), together with the wide diversity of mutations in the 1830-bp coding region of the *MEN1* gene, makes mutational analysis for diagnostic purposes in MEN1 more difficult than that for MEN2 (Thakker, 1998).

A total of 24 different polymorphisms (12 in the coding region [10 synonymous and 2 non synonymous], 9 in the introns, and 3 in the untranslated regions) of the *MEN1* gene have been reported (Fig. 3 and Table 4) (Lemos and Thakker, 2008). It is important to recognize the occurrence of these polymorphisms as they need to be distinguished from mutations when performing analysis for genetic diagnosis.

More than 90% of tumors from MEN1 patients have loss of heterozygosity (LOH), and this has generally been taken as evidence that the *MEN1* gene acts as a tumor-suppressor gene, consistent with Knudson’s two-hit hypothesis (Friedman et al., 1989; Larsson et al., 1988; Thakker et al., 1989). However, this LOH represents only one mechanism by which the second hit may occur, with the other mechanisms being intragenic deletions and point mutations. MEN1 tumors (e.g., parathyroids, insulinoma, and lipoma) that do not have LOH have been shown to harbor different somatic and germ-line mutations of the *MEN1* gene (Fig. 4) (Pannett and Thakker, 2001), and this is consistent with the Knudson two-hit hypothesis (Knudson et al., 1973).

### 2.4 MEN1 mutations in sporadic non-MEN1 endocrine tumors

Parathyroid, pancreatic islet cell, and anterior pituitary tumors may occur either as part of MEN1 or more commonly as sporadic, nonfamilial tumors. Tumors from patients with MEN1 have been observed to harbor the germ-line mutation together with a somatic LOH involving chromosome 11q13 (Friedman et al., 1989; Larsson et al., 1988; Thakker et al., 1989, 1993), or point mutations, as expected from Knudson’s model (Pannett and Thakker, 2001) and the proposed role of the *MEN1* gene as a tumor suppressor. However, LOH involving chromosome 11q13, which is the location of *MEN1*, has also been observed in 5–50% of sporadic endocrine tumors, thus implicating the *MEN1* gene in the etiology of these tumors (Thakker et al., 1993). Approximatley 200 somatic *MEN1* mutations (Fig. 4) have been reported between 1997 and 2007, and these have occurred in several different endocrine tumors (Lemos and Thakker, 2008). Thus, these have been detected in 18% of sporadic parathyroid tumors (total number, *n* = 452), 38% of gastrinomas (*n* = 105) (Wang et al., 1998), 14% of insulinomas (*n* = 43), 57% of VIPomas (*n* = 7) (Wang et al., 1998), 16% of non-functioning pancreatic tumors (*n* = 32), 60% of glucagonomas (*n* = 5), 2.0% of adrenal cortical tumors (*n* = 83) (Lemos and Thakker, 2008), 35% of bronchial carcinoid tumors (*n* = 26) (Lemos and Thakker, 2008), 3.5% of anterior pituitary adenomas (*n* = 167) (Lemos and Thakker, 2008; Prezant et al., 1998), 10% of angiofibromas (*n* = 19), and 28% of lipomas (*n* = 8). These somatic mutations are scattered throughout the 1830-bp coding region (Fig. 3), and 18% are nonsense mutations, 40% are frameshift deletions or insertions, 6% are in-frame deletions or insertions, 7% are splice-site mutations, and 29% are missense mutations (Fig. 4) (Lemos and Thakker, 2008). A comparison of the locations of the somatic and germ-line mutations revealed a higher frequency (39% [somatic] vs. 23% [germline]; *P* < 0.001) of somatic mutations in exon 2, but the significance of this observation (Lemos and Thakker, 2008) in the context of the Knudson two-hit hypothesis remains to be elucidated. The tumors harboring a somatic *MEN1* mutation had chromosome 11q13 LOH as the other genetic abnormality, or “hit”, consistent with Knudson’s hypothesis. These studies (Lemos and Thakker, 2008; Prezant et al., 1998; Wang et al., 1998) indicate that although inactivation of the *MEN1* gene may have a role in the etiology of some sporadic endocrine tumors, the involvement of other genes, for example, the GNAS1 gene encoding the G protein-stimulatory α subunit (Lyons et al., 1990), with major roles in the etiology of such sporadic endocrine tumors, is highly likely.

### 2.5 MEN1 mutations in hereditary endocrine disorders

The role of the *MEN1* gene in the etiology of other inherited endocrine disorders, in which either parathyroid or pituitary tumors occur as an isolated endocrinopathy, has been investigated by mutational analysis. *MEN1* mutations have been reported in 42 families with isolated hyperparathyroidism (FIHP) (Hannan et al., 2008; Lemos and Thakker, 2008), and 38% of these are missense mutations, and fewer than 31% are nonsense or frame-shift mutations, which would result in a truncated and likely inactivated protein. This contrasts significantly (*P* < 0.01) with the situation in MEN1 patients in whom more than 65% of the germ-line mutations are protein-truncation and about 23% are missense mutations (Fig. 4). These observations are consistent with a more likely association between missense mutations and the milder FIHP variant, but it is important to note that the mutations associated with FIHP are also scattered throughout the coding region and not clustered, a situation that is similar to that found for germ-line *MEN1* mutations (Fig. 3). Furthermore, the occurrence of protein-truncating mutations in FIHP patients and particularly deletions, such as the 4 bp, involving codons 83–84 (Table 2), which are identical to those observed in MEN1 patients, makes it difficult to establish an unequivocal phenotype–genotype correlation. However, the sole occurrence of parathyroid tumors in these families that harbor *MEN1* mutations that are similar to those found in families with MEN1 is remarkable, and the mechanisms that determine the altered phenotypic expressions of these mutations remain to be elucidated. However, nonsense mutations (Tyr312Stop and Arg460Stop) have been detected (Olufemi et al., 1998) in MEN1 families with the Burin or prolactinoma variant (Agarwal et al., 1997) which is characterized by a high occurrence of prolactinomas and a low occurrence of gastrinomas (Hao et al., 2004; Olufemi et al., 1998). Furthermore, a splice mutation (c.446-3c → g) has been detected in an MEN1 kindred from Tasmania, in whom there was an absence of somatotrophinomas (Burgess et al., 2000). However, some other families with isolated acromegaly do not have abnormalities of the *MEN1* gene (Lemos and Thakker, 2008), even though segregation analysis in families with isloated acromegaly and LOH studies of somatotrophinomas indicated that the gene was likely to be located on chromosome 11q13 (Soares et al., 2005). Interestingly, mutations of the aryl hydrocarbon receptor interacting protein (*AIP*) gene, which is also located on chromosome 11q13 and 2.7 Mb telomeric to the *MEN1* gene, have been identified in some families with isolated acromegaly (Vierimaa et al., 2006). However *AIP* mutations have not been deteced in patients with MEN1, who do not have *MEN1* mutations.

### 2.6 Genetic testing and screening in MEN1

MEN1 is an uncommon disorder, but because of its autosomal-dominant inheritance, the finding of MEN1 in a patient has important implications for other family members; first-degree relatives have about a 50% risk of development of the disease (Trump et al., 1996). Screening for MEN1 in patients involves the detection of tumors and ascertainment of the germ-line genetic state, that is, normal or mutant gene carrier (Fig. 1) (Thakker et al., 2012). Detection of tumors entails clinical, biochemical, and radiologic investigations for MEN1-associated tumors in patients (Trump et al., 1996). The characterization of the *MEN1* gene (Chandrasekharappa et al., 1997; The European Consortium on MEN1, 1997) has facilitated identification of individuals who have mutations and hence a high risk of acquiring the disease (Figs. 2 and 3).

#### 2.6.1 MEN1 mutational analysis

*MEN1* mutational analysis is helpful in clinical practice in several ways that include: (1) confirmation of the clinical diagnosis; (2) identification of family members who harbor the *MEN1* mutation and require screening for tumor detection and early treatment; and (3) identification of the 50% of family members who do not habour the familial germline *MEN1* mutation and can therefore be reassured and alleviated of the burden of anxiety of developing tumors. This latter aspect cannot be over-emphasized as it not only helps to reduce the personal cost to the individuals and their children, but also to the health services in not having to undertake unnecessary biochemical and radiological investigations (Table 5). Thus, *MEN1* mutational analysis can be useful in clinical practice.

The current guidelines recommend that *MEN1* mutational analysis should be undertaken in: (1) an index case with two or more MEN1-associated endocrine tumors (i.e. parathyroid, pancreatic or pituitary tumors); (2) asymptomatic first degree relatives of a known *MEN1* mutation carrier; (3) a first-degree relative of a *MEN1* mutation carrier expressing familial MEN1 (i.e. having symptoms, signs, biochemical or radiological evidence for one or more MEN1-associated tumors) (Thakker et al., 2012). In addition, *MEN1* mutational analysis should be considered in patients with suspicious or atypical MEN1. This would include individuals with: parathyroid adenomas before the age of 30 years or multigland parathyroid disease; gastrinoma or multiple pancreatic islet cell tumors at any age; or individuals who have two or more MEN1-associated tumors, which are not part of the classical triad of parathyroid, pancreatic islet and anterior pituitary tumors (e.g. parathyroid tumor plus adrenal tumor) (Thakker et al., 2012).

Family members, including asymptomatic individuals who have been identified to harbor a *MEN1* mutation will require biochemical and radiological screening (5). In contrast, those relatives who do not harbor the *MEN1* mutation will have their risk of developing MEN1-associated endocrine tumors markedly decreased from 1 in 2 for an autosomal dominant disorder, to 1 in 3000, 1 in ∼100,000 and 1 in 1000 which are the respective risks for parathyroid, pancreatic islet cell and anterior pituitary tumors for the general population; thus, these relatives without the *MEN1* mutation will be freed from the requirement of further repeated clinical investigations (Lemos and Thakker, 2008; Thakker et al., 2012).

*MEN1* germline mutational analysis should be considered in those presenting at an early age with a single, apparently sporadic MEN1-associated tumor. The occurrence of germline *MEN1* mutations in all patients with sporadic, non-familial parathyroid adenomas is 1%, in gastrinomas is 5%, in prolactinoma is 1%, and in foregut carcinoids is 2% (Thakker et al., 2012). However, the occurrence of *MEN1* mutations tumors in patients developing parathyroid tumors below the age of 40 years has been reported to be higher at 5–13%. The occurrence rates of germline *MEN1* mutations in individuals presenting with a single apparent sporadic pancreatic NET at similarly younger age, has not been established, and the current guidelines recommend that *MEN1* mutational analysis should be considered in those with gastrinoma or multiple pancreatic NETS (Thakker et al., 2012).

Mutational analysis in asymptomatic individuals should be undertaken at the earliest opportunity, and where possible in the first decade of life as tumors have developed in some children by the age of 5 years (Newey et al., 2009; Stratakis et al., 2000; Thakker et al., 2012). Appropriate biochemical and radiological investigations aimed at detecting the development of tumors should then be undertaken in these individuals (Tables 1 and 5). The current guidelines recommend that these investigations should commence from the age of 5 years to detect anterior pituitary tumors and insulinomas, the age of 8 years to detect parathyroid tumors, and the age of 20 years to detect gastrinomas and foregut carcinoid tumors (Table 5), and be undertaken thereafter on an annual basis (Thakker et al., 2012).

### 2.7 Detection of MEN1 tumors

**Fig. 5:**
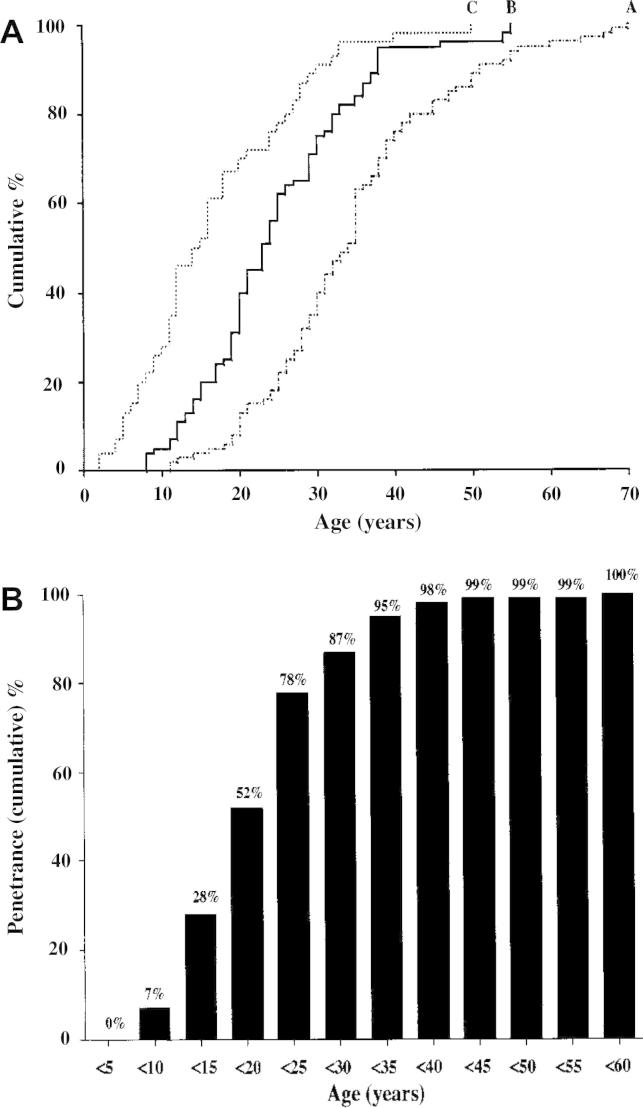
Age distributions (A) and age-related penetrance (B) of multiple endocrine neoplasia type 1 (MEN1) determined from an analysis of 174 mutant gene carriers. A, The age distributions were determined for three groups of MEN1 mutant gene carriers from 40 families in whom mutations were detected (Bassett et al., 1998). The 91 members of group A had symptoms, whereas the 40 members of group B were asymptomatic and detected by biochemical screening. The 43 members of group C represent individuals who are MEN1 mutant gene carriers (see Fig. 2) and who remain asymptomatic and biochemically normal. The ages included for members of groups A, B, and C are those at the onset of symptoms, at the finding of the biochemical abnormality, and at the last clinical and biochemical evaluation, respectively. Groups B and C contained members who were significantly younger than those in group A (*P* < 0.001). The younger age of the group C mutant gene carriers is consistent with an age-related penetrance for MEN1, which was calculated (B) for the first five decades. The age-related penetrance (i.e., the proportion of mutant gene carriers with manifestations of the disease by a given age) increased steadily from 7% in the group younger than 10 years to 52%, 87%, 98%, 99%, and 100% by the ages of 20, 30, 40, 50, and 60 years, respectively. The residual risk (100% – age-related penetrance) for the development of MEN1 tumors in asymptomatic mutant gene carriers who are biochemically normal would then be 93%, 48%, 13%, 2%, and 1% at the ages of 10, 20, 30, 40, and 50 years, respectively. (From Bassett JHD, Forbes SA, Pannett AAJ, et al.: Characterization of mutations in patients with multiple endocrine neoplasia type 1 (MEN1). *Am J Hum Genet* 62: 232–244, 1998, with permission.) (Bassett et al., 1998).

Biochemical screening for the development of MEN1 tumors in asymptomatic members (Fig. 2) of families with MEN1 is of great importance in as much as earlier diagnosis and treatment of these tumors may help reduce morbidity and mortality (Thakker et al., 2012). The age-related penetrance (i.e., the proportion of gene carriers manifesting symptoms or signs of the disease by a given age) has been ascertained (Bassett et al., 1998) (Fig. 5), and the mutation appears to be nonpenetrant in those younger than 5 years. Thereafter, the mutant MEN1 gene has a high penetrance, being greater than 50% penetrant by 20 years of age and greater than 95% penetrant by 40 years (Bassett et al., 1998). Screening for MEN1 tumors is difficult because the clinical and biochemical manifestations in members of any one family are not uniformly similar (Thakker et al., 1989; Trump et al., 1996). Attempts to screen for the development of MEN1 tumors in the asymptomatic relatives of an affected individual have depended largely on measuring the serum con centrations of calcium, gastrointestinal (g–i) hormones (e.g., gastrin), and prolactin, as well as on radiologic imaging of the abdomen and pituitary (Thakker et al., 1989; Thakker, 1998). Parathyroid overactivity causing hypercalcemia is almost invariably the first manifestation of the disorder and has become a useful and easy biochemical screening investigation (Trump et al., 1996). In addition, hyperprolactinemia, which may be asymptomatic, may represent the first manifestation in fewer than 10% of patients and may thus also be a useful and easy biochemical screening investigation (Thakker et al., 2012; Trump et al., 1996). Pancreatic involvement in asymptomatic individuals has been detected by estimating the fasting plasma concentrations of gastrin, PP, and glucagon and by abdominal imaging (Scarsbrook et al., 2006; Thakker et al., 2012; Trump et al., 1996). However, one study has shown that a stimulatory meal test is a better method for detecting pancreatic disease in individuals who have no demonstrable pancreatic tumors by CT. An exaggerated increase in serum gastrin or PP or both proved to be a reliable early indicator for the development of pancreatic tumors in these individuals.

**Fig. 6:**
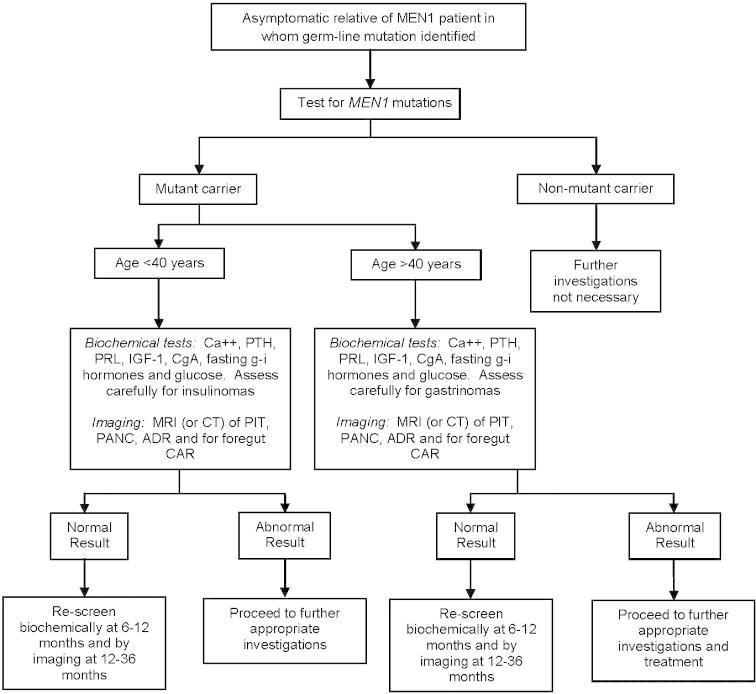
An approach to screening in an asymptomatic relative of a patient with multiple endocrine neoplasia type 1 (MEN1). The relative should have first undergone clinical evaluation for MEN1-associated tumors to establish that the individual is asymptomatic. Relatives who are symptomatic, who should also have a test for MEN1 mutations, should proceed to appropriate investigations and management. If mutational analysis for MEN1 is not available, then this protocol could be adapted for first-degree relatives (Trump et al., 1996). The use of mutational analysis and such screening methods in children is controversial and varies in different countries. It has been suggested that nonessential genetic testing in a child who is not old enough to make important long-term decisions be deferred. However, the finding that a child from a family with MEN1 does not have any *MEN1* mutations removes the burden of repeated clinical, biochemical, and radiologic investigations and enables health resources to be more effectively directed toward those children who are *MEN1* mutant gene carriers. The approaches to genetic testing and screening in MEN1 vary in different countries. PIT, pituitary; PANC, pancreas; ADR, adrenal; CAR, carcinoid; PTH, parathyroid hormone; PRL, prolactin; IGF-1, insulin-growth-factor-1; CgA, chromogranin A; and g–i, and gastro-intestinal gut-hormones. (Reproduced with permission from Thakker RV (2010) Multiple Endocrine Neoplasia Type 1. In *Endocrinology*, sixth ed., Eds: LJ De Groot, JL Jameson.) (Thakker, 2010).

At present, it is suggested that individuals at high risk for MEN1 (i.e., mutant gene carriers) undergo biochemical screening (Fig. 6) at least once per annum and also have baseline pituitary and abdominal imaging (e.g., MRI or CT), which should then be repeated at 1- to 3-year intervals (Table 5) (Thakker et al., 2012). Screening should commence in early childhood because the disease has developed in some individuals by the age of 5 years (Thakker et al., 2012), and it should continue for life because the disease has not developed in some individuals until the eighth decade. The screening history and physical examination should be directed toward eliciting the symptoms and signs of hypercalcemia, nephrolithiasis, peptic ulcer disease, neuroglycopenia, hypopituitarism, galactorrhea and amenorrhea in women, acromegaly, Cushing’s disease, and visual field loss and the presence of subcutaneous lipomas, angiofibromas, and collagenomas (Thakker et al., 2012; Trump et al., 1996). Biochemical screening should include estimations of serum calcium, PTH, gastrointestinal hormones (e.g., gastrin, insulin with a fasting glucose, glucagon, VIP, and PP), chromogranin A, prolactin, and IGF-1 in all individuals (Thakker et al., 2012), and more specific endocrine-function tests should be undertaken in individuals who have symptoms or signs suggestive of a clinical syndrome. Radiologic screening should include an MRI (or CT scanning) of the pancreas, adrenals, and pituitary, initially as a baseline and then every 1–3 years, as well as imaging for foregut carcinoids (Scarsbrook et al., 2006; Thakker et al., 2012).

### 2.8 MEN1 phenocopies

Approximately 5–25% of patients with MEN1 may not have mutations of the *MEN1* gene. This variability in detecting *MEN1* mutations may partly be attributable to differences in methods used to identify the mutations; for example most studies do not systematically examine for large gene deletions, which may be found in up to 33% of patients who do not have coding region mutations (Cavaco et al., 2002). In addition, this variability may be due to phenotype ascertainment, as some studies have included non-familial (i.e. sporadic) patients who may have developed only two (or fewer) endocrine tumors, and the detection rate for *MEN1* mutations in these patients was found to be <5% (Tham et al., 2007). Such patients with MEN1-associated tumors but without *MEN1* mutations may represent phenocopies, or have mutations involving other genes. Phenocopy refers to the development of disease manifestations usually associated with mutations of a particular gene, but instead are due to another etiology, and the occurrence of phenocopies, has been reported in 5–10% of MEN1 kindreds (Burgess et al., 2000; Newey and Thakker, 2011; Turner et al., 2010). These phenocopies occurred in two settings. Firstly, in the context of familial MEN1 (Fig. 1), in which a patient with one MEN1-associated tumor, e.g. a prolactinoma, did not have the familial mutation; and secondly, in the context of clinical MEN1, in which patients with two MEN1-associated tumors, who did not have an *MEN1* mutation, were demonstrated to have involvement of other genes. These genes may include: *CDC73*, which encodes parafibromin, whose mutations result in the Hyperparathyroid–jaw tumor (HPT–JT) syndrome; the calcium sensing receptor (CaSR), whose mutations result in familial benign hypercalciuric hypercalcemia (FBHH) (Turner et al., 2010); and the aryl hydrocarbon receptor-interacting protein (*AIP*), a tumor suppressor located on chromosome 11q13 whose mutations are associated with familial isolated pituitary adenomas (FIPA) (Belar et al., 2012). The occurrence of MEN1 phenocopies may confound the diagnosis of MEN1 (Fig. 1) in an individual, and it therefore appears advisable to offer genetic testing to determine the *MEN1* mutation status to symptomatic family members within a MEN1 kindred, as well as to all index cases (i.e. patient) with two or more endocrine tumors. If a *MEN1* mutation is not identified in the index case with two or more endocrine tumors, then clinical and genetic tests for other disorders such as HPT–JT, FBHH, or FIPA should be considered, as these patients may represent phenocopies for MEN1.

## 3 Function of menin

Initial analysis of the predicted amino acid sequence of menin did not reveal homologies to any other proteins, sequence motifs, signal peptides, or consensus nuclear localization signal (Chandrasekharappa et al., 1997), and thus the putative function of menin could not be deduced. However, studies based on immunofluorescence, Western blotting of subcellular fractions, and epitope tagging with enhanced green fluorescent protein revealed that menin was located primarily in the nucleus (Guru et al., 1998). Menin has been shown to have at least three nuclear localization signals (NLSs) that are located in the C-terminal quarter of the protein (Guru et al., 1998; La et al., 2006) (Fig. 3). Interestingly, the truncated MEN1 proteins that would result from the nonsense and frameshift mutations, if expressed, would lack at least one of these nuclear localization signals (Fig. 3). Menin is predominantly a nuclear protein in non-dividing cells, but in dividing cells, it is found in the cytoplasm (Guru et al., 1998). The function of menin still remains to be elucidated, but it has been shown to interact with a number of proteins that are involved in transcriptional regulation (Agarwal et al., 1999; Dreijerink et al., 2006; Heppner et al., 2001; Hughes et al., 2004; Karnik et al., 2005; La et al., 2007; Milne et al., 2005; Roudaia and Speck, 2008; Scacheri et al., 2006; Yokoyama and Cleary, 2008; Yokoyama et al., 2004), genome stability, cell division and proliferation (Lemos and Thakker, 2008) (Fig. 3 and Table 6). Thus, in transcriptional regulation, menin has been shown to interact with: the activating protein-1 (AP-1), transcription factors JunD (Agarwal et al., 1999) and C-Jun (Yumita et al., 2003), and to suppress Jun-mediated transcriptional activation; members (e.g., p50, p52, and p65) of the NF-_К_B family of transcriptional regulators to repress NF-_К_B-mediated transcriptional activation (Heppner et al., 2001); members of the Smad family, Smad3 and the Smad 1/5 complex, to inhibit the transforming growth factor-β (TGF-β) and the bone morphogenetic protein-2 (BMP-2) signaling pathways, respectively; Runx2, also called cbfa1, which is a common target of TGF-β and BMP-2 in differentiating osteoblasts (Sowa et al., 2004); and the mouse placental embryonic (pem) expression gene, which encodes a homeobox-containing protein. Recently, the forkhead transcription factor CHES1 has been shown to be a component of this transcriptional repressor complex and to interact with menin in an S-phase checkpoint pathway related to DNA damage response. Menin uncouples ELK-1, JunD, and c-Jun phosphorylation from mitogen-activated protein kinase (MAPK) activation and suppresses insulin-induced c-Jun-mediated transactivation in CHO-1R cells.

Menin has also been reported to directly bind to doubled-stranded DNA and this is mediated by the positively charged residues in the nuclear localization signals (NLSs) in the carboxyl terminus of menin. The NLSs appear to be necessary for menin to repress the expression of the insulin-like growth factor binding protein-2 (IGFBP-2) gene by binding to the IGFBP-2 promoter. In addition, each of the NLSs has also been reported to be involved in menin-mediated induction of caspase 8 expression (La et al., 2006, 2007). The NLSs may therefore have roles in controlling gene transcription as well as targeting menin into the nucleus. Furthermore, gene expression profile studies, using pituitary and pancreatic islet tumors obtained from *men1* mouse models, have revealed altered expression of genes involved in transcription, cell cycle and chromatin remodeling.

A role for menin in controlling genome stability has been proposed because of its interactions with: a subunit of replication protein (RPA2), which is a heterotrimeric protein required for DNA replication, recombination, and repair; and the FANCD2 protein, that is involved in DNA repair and mutations of which result in the inherited cancer-prone syndrome of Fanconi’s anemia. Menin also has a role in regulating cell division as it interacts with the nonmuscle myosin II-A heavy chain (NMHC II-A), which participates in mediating alterations in cytokinesis and cell shape during cell division and the glial fibrillary acidic protein (GFAP) and vimentin, which is involved in the intermediate filament network. Menin also has a role in cell cycle control as it interacts with: the tumor metastases suppressor NM23H1/nucleoside diphosphate kinase, which induces guanosine triphosphate (GTP)ase activity; and the activator of S-phase kinase (ASK), which is a component of the Cdc7/ASK kinase complex that is crucial for cell proliferation. Indeed, menin has been shown to completely repress ASK-induced cell proliferation (Schnepp et al., 2004).

The functional role of menin as a tumor suppressor also has been investigated, and studies in human fibroblasts have revealed that menin acts as a repressor of telomerase activity via hTERT (a protein component of telomerase) (Lin and Elledge, 2003). Furthermore, overexpression of menin in the human endocrine pancreatic tumor cell line (BONI) resulted in an inhibition of cell growth (Stalberg et al., 2004) that was accompanied by upregulation of JunD expression but downregulation of δ-like protein 1/preadipocyte factor-1, proliferating cell nuclear antigen, and QM/Jif-1, which is a negative regulator of C-Jun (Stalberg et al., 2004). These findings of growth suppression by menin also are observed in other cell types. Thus, expression of menin in RAS-transformed NIH3T3 cells partially suppressed the RAS-mediated tumor phenotype in vitro and in vivo (Stalberg et al., 2004), and overexpression of menin in CHO-IR cells also suppressed insulin-induced AP-1 transactivation, and this was accompanied by an inhibition of C-Fos induction at the transcriptional level (Kim et al., 1999). Furthermore, menin expression in *Men1*-deficient mouse Leydig tumor cell lines induced cell cycle arrest and apoptosis (Hussein et al., 2007). In contrast, depletion of menin in human fibroblasts resulted in their immortalisation (Lin and Elledge, 2003). Thus, menin appears to have a large number of functions through interactions with proteins, and these mediate alterations in cell proliferation.

### 3.1 Menin and epigenetic regulation

**Fig. 7:**
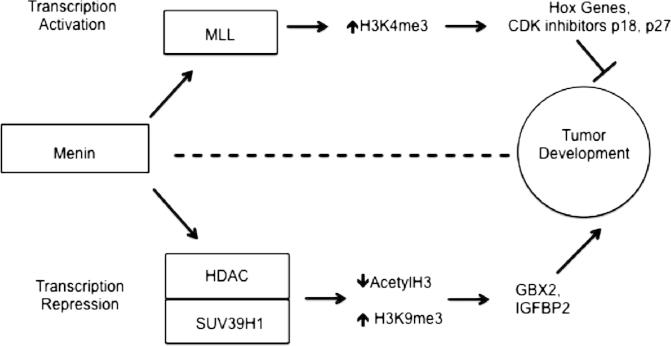
Role of menin in regulating transcription by epigenetic mechanisms. Menin may act as an activator or repressor of transcription depending on its inteaction with the MLL complex, HDAC or SUV39H1. Thus, interaction with MLL results in methylation of histone H3 (H3K4), which in turn regulates suppression of the CDK inhibitors p18 and p27, and Hox genes, to suppress cell proliferation. However, interaction with HDAC and SUV39H1 results in acetylation of H3 and methylation of H3K9me3, respectively, which in turn regulate expression of GBX2 and IGFBP2 to promote cell proliferation. Thus, menin plays a role in selective mediation of chromatin remodeling and thereby in regulating gene expression and cell proliferation.

Menin which acts as a scaffold protein, may increase or decrease gene expression by epigenetic regulation of gene expression via histone methylation or acetylation. For examaple, menin has been shown to be an integral component of histone methyltransferase complexes that contain members from the mixed-lineage leukemia (MLL) and trithorax protein family (Hughes et al., 2004). These can methylate the lysine 4 residue of histone H3 (H3K4) and H3K4 trimethylation is linked to activation of transcription. Menin, by serving as a molecular adaptor, physically links the MLL histone methyl transferase with the lens epithelium-derived growth factor (LEDGF), which is a chromatin-associated protein implicated in the eitology of leukemia, autoimmunity and human immuno-defeciency virus-1 (HIV-1) disease (Roudaia and Speck, 2008; Yokoyama and Cleary, 2008). Thus, menin has a critical function in the MLL complex and its regulation of multiple transcriptional pathways, for example, menin, as a component of this MLL complex, regulates the expression of genes such as the *Hox* homeobox genes (Agarwal and Jothi, 2012; Yokoyama et al., 2004) and the genes for cyclin-dependent kinase inhibitors, p27 and p18 (Karnik et al., 2005; Milne et al., 2005). Interestingly, specific deregulation of a subset of 23 Hox genes has been reported in parathyroid tumors from patients with MEN1 (Shen et al., 2008). In addition, menin has been shown to directly interact with protein arginine methyltransferase 5 (PRMT5), and to recruit PRMT5 to the promoter of the gene encoding the growth arrest-specific protein 1 (GAS1), which is an important factor for binidng of Sonic Hedgehog (Shh) ligand to its cell surface receptor patched (PTCH1) with subsequent activation of the Hedgehog signaling pathway, which increases repressive histone arginine symmetric dimethylation (H4R3M2S) and suppresses GAS1 expression (Gurung et al., 2013). GAS1 blocks entry to S phase and prevents cycling of normal and transformed cells, hereby acting as a tumor suppressor. Furthermore, menin has been reported to interact with the suppressor of varigation 3–9 homolog family protein (SUV39H1) to mediate H3K mehylation, and thereby silence transcrpitional activty of target genes (Yang et al., 2013). Menin has been shown to directly interact with the nuclear receptor for estrogen (ERα) and to act as a coactivator for ERα–mediated transcription, linking the activated estrogen receptor to histone H3K4 trimethylation (Dreijerink et al., 2006). Additional studies have also shown that the interaction of menin with JunD may be mediated by a histone deacetylase-dependent mechanism, via recruitment of an mSin3A-histone deacetylase (HDAC) complex to repress JunD transcriptional activity (Kim et al., 2003, 2005). Thus, these studies reveal that menin can act both as an activator and repressor of transcription by epigenetic mechanisms that involve integration of different chromatin modification complexes. For example, menin may mediate repression of genes targeted by the transcription factor JunD through the mSin3A-histone deacetylse (HDAC) complex, and the growth factor pleiotrophin gene through polycomb group complex-mediated histone H3K27 methylation (Gao et al., 2009). Menin may also promote expression of genes, such as those encoding the cyclase-dependent-kinase (CDK) inhibitors, p27^Kip1^ and P18^INK4c^, via interaction with the MLL-HMT complex that mediates histone H3K4 methylation (Fig. 7) (Huang et al., 2012; Hughes et al., 2004; Karnik et al., 2005; Milne et al., 2005). Targeting of these epigenetic mechanisms by use of small molecules is providing important insights for the development of therapeutic compounds. For example, the crystal structures of human menin in its free form and in complexes with MLL1 have shown that menin contains a deep pocket that binds to a short peptide of MLL1 (Huang et al., 2012). High-throughput screening studies to identify compounds that target menin and suppress its interaction with MLL have identified that thienopyrimidine has such functions (Grembecka et al., 2012; McCarthy, 2012; Shi et al., 2012). Furthermore, their structure activity analyses led to generation of some thienopyrimidine analogs, which bind to wild-type menin but not menin mutants that involve the interaction site with MLL, and others which are capable of inhibiting the MLL-fusion protein-mediated leukemogenic transformation (Grembecka et al., 2012; McCarthy, 2012; Shi et al., 2012). Thus, understanding the role of menin in epigenetic mechanisms has provided a rational and molecular basis for the development of menin-MLL inhibitors.

## 4 Multiple endocrine neoplasia type 4 (MEN4)

Approximately 5–10% of patients with MEN1 do not have mutations of the *MEN1* gene (Lemos and Thakker, 2008; Thakker et al., 2012), and these patients may have mutations involving other genes. One of these genes is the *CDNK1B*, which encodes the 196 amino acid cyclin-dependent kinase inhibitor (CK1) p27^kip1^ and was identified to be involved by investigations of a recessive MEN-like syndrome in a naturally occuring rat model, referred to as MENX (Pellegata et al., 2006). Rats with MENX were observed to develop parathyroid adenomas, pancreatic islet-cell hyperplasia, thyroid C-cell hyperplasia, bilateral phaeochromocytomas, paragangliomas and cataracts. The disorder was inherited as an autosomal recessive trait. Genetic mapping studies localized *MENX* to the distal part of rat chromosome 4, a region that also contained the putative tumor suppressor CK1P27kip1, which is also refered to as p27. Mutational analysis of the *CDNK1B* gene identified, in rats with MENX, a homozygous frameshifting insertion of 8 bp, at codon 177, that resulted in a missense peptide and termination at codon 218 (Pellegata et al., 2006). This *CDNK1B* mutation resulted in an absence of p27 protein in the tumor cells (Pellegata et al., 2006). These findings prompted studies in patients with MEN1, who did not harbor *MEN1* mutations, for abnormalities of *CDKN1B* which in man is located on chromosome 12p13. This revealed that approximatley 3% of these patients with MEN1-associated tumors, such as parathyroid adenomas, pituitary adenomas and pancreatic NETs in association with gonadal, adrenal, renal and thyroid tumors have *CDNK1B* mutations. To date, 8 different heterozygous loss-of-function *CDNK1B* mutations have been identified in patients with MEN1-like tumors, and this indicates that MEN4 in man is an autosomal dominant-disorder, unlike MENX in rats which is autosomal recessive. In addition, germline *CDNK1B* mutations may also, rarely, be found in patients with sporadic (i.e. non-familial) forms of primary hyperparathyroidism.

## 5 Conclusions

Combined clinical and laboratory investigations of MEN1 have resulted in an increased understanding of this disorder, which may be inherited as an autosomal-dominant condition. Defining the features of each disease manifestation in MEN1 has improved patient management and treatment and has also facilitated a screening protocol to be instituted. Application of the techniques of molecular biology has enabled identification of the gene causing MEN1 and detection of mutations in patients. In addition, these recent advances have facilitated the identification of mutant *MEN1* gene carriers who are at high risk for development of this disorder and thus require regular and biochemical and radiologic screening to detect the development of endocrine tumors. The function of the protein encoded by the *MEN1* gene, menin, has been shown to involve regulation of transcription, which involves genetic mechanisms, genome stability, and cell division, but much still remains to be elucidated. Furthermore, inhibitors of the menin-MLL interaction hold promise for provoking new treatments.

## Figures and Tables

**Table 1:** Multiple endocrine neoplasia syndromes and their characteristic tumors and associated genetic abnormalities.

Type (chromosome location)	Tumors (estimated penetrance)	Gene; most frequently mutated codons
MEN1 (11q13)	Parathyroid adenoma (90%)	*MEN1*

	Entero-pancreatic tumor (30–70%)	83/84, 4-bp del (≈4%)
	– Gastrinoma (40%)	119, 3-bp del (≈3%)
	– Insulinoma (10%)	209–211, 4-bp del (≈8%)
	– Non-functioning & PPoma (20–55%)	418, 3-bp del (≈4%)
	– Glucagonoma (<1%)	514–516, del or ins (≈7%)
	– VIPoma (<1%)	Intron 4 ss, (≈10%)

	Pituitary adenoma (30–40%)	
	– Prolactinoma (20%)	
	– Somatotrophinoma (10%)	
	– Corticotrophinoma (<5%)	
	– Non-functioning (<5%)	

	Associated Tumors/	
	– Adrenal cortical tumor (40%)	
	– Phaeochromocytoma (<1%)	
	– Brochopulmonary NET (2%)	
	– Thymic NET (2%)	
	– Gastric NET (10%)	
	– Lipomas (30%)	
	– Angiofibromas (85%)	
	– Collagenomas (70%)	
	– Meningiomas (8%)	
		

*MEN2 (10 cen-10q11.2)*		
MEN2A	MTC (90%)	*RET*
	Phaeochromocytoma (50%)	634, missense
	Parathyroid adenoma (20–30%)	e.g. Cys → Arg (∼85%)

MTC only	MTC (100%)	*RET*
		618, missense (>50%)

MEN2B (also known as MEN3)	MTC (>90%)	*RET*
	Phaeochromocytoma (40–50%)	918, Met → Thr (>95%)
	Associated abnormalities (40–50%)	
	Mucosal neuromas	
	Marfanoid habitus	
	Medullated corneal nerve fibers	
	Megacolon	

MEN4 (12p13)	Parathyroid adenoma^a^	*CDKN1B*
	Pituitary adenoma^a^	No common mutations identified to date
	Reproduction organ tumors^a^ (e.g. testicular cancer, neuroendocrine cervical carcinoma)	
	Adrenal + renal tumors^a^	

Autosomal-dominant inheritance of the MEN syndromes has been established. del, deletion; ins, insertion; PPoma, pancreatic polypeptide–secreting tumor; VIPoma, vasoactive intestinal polypeptide–secreting tumor; MTC, Medullary Thyroid Cancer. Adapted from Thakker RV: Clinical Practice Guidelines for Multiple Endocrine Neoplasia Type 1 (MEN1). *J Clin Endocrinol Metab* 97: 2990–3011, ©2012. The Endocrine Society (Thakker et al., 2012).

a Insufficient numbers reported to provide prevalence information.

**Table 2:** *MEN1* germline mutations occurring in over 1.5% of affected families.

Mutation^a^	DNA sequence change^b^	Exon	Codon	Predicted effect^c^	Frequency (%)^d^
I	c.249_252delGTCT	2	83–84	fs	4.5
II	c.292C>T	2	98	ns, Arg98Stop	1.5
III	c.358_360delAAG	2	120	if	1.7
IV	c.628_631delACAG	3	210–211	fs	2.5
V	c.784-9G>A	Intron 4	–	sp	1.9
VI	c.1243C>T	9	415	ns, Arg415Stop	1.5
VII	c.1378C>T	10	460	ns, Arg460Stop	2.6
VIII	c.1546delC	10	516	fs	1.8
IX	c.1546_1547insC	10	516	fs	2.7

a Mutation number as referred to in Fig. 3.

b Mutations are numbered in relation to the *MEN1* cDNA reference sequence (GenBank accession number NM_130799.1), whereby nucleotide +1 corresponds to the A of the ATG-translation initiation codon.

c fs – frameshift mutation; ns – nonsense mutation; sp – splice site mutation; if – in-frame mutation.

d Frequencies based on 1133 reported *MEN1* independent germline mutations. (From Lemos M and Thakker RV: Multiple Endocrine Neoplasia Type 1 (MEN1): Analysis of 1336 Mutations Reported in the First Decade Following Identification of the Gene. *Hum Mutat* 29: 22–32, 2008, with permission) (Lemos and Thakker, 2008).

**Table 3:** Multiple endocrine neoplasia type 1 – associated tumors in five unrelated families with a 4-bp deletion at codons 210 and 211.

Tumors	Family				
	1	2	3	4	5
Parathyroid	+	+	+	+	+
Gastrinoma	+	−	+	+	+
Insulinoma	−	+	−	−	−
Glucagonoma	−	−	−	−	+
Prolactinoma	−	+	+	+	+
Carcinoid	+	−	−	−	−

+, Presence; −, Absence of tumors. Adapted from Thakker RV: Multiple endocrine neoplasia—syndromes of the twentieth century. *J Clin Endocrinol Metab* 83: 2617–2620, ©1998. The Endocrine Society (Thakker, 1998).

**Table 4:** Polymorphisms of the *MEN1* gene.

Polymorphism^a^	DNA sequence change^b^	Exon	Codon change	Allele frequency^c^
a	c.-533T>A	1	–	0.32
b	c.-533T>C	1	–	0.12
c	c.-39C>G	Intron 1	–	0.20
d	n/a	2	Leu10Leu	n/a
e	c.435C>T	2	Ser145Ser	0.01
f	c.445+183G>A	Intron 2	–	0.05
g	c.446−127A>T	Intron 2	–	n/a
h	c.446−58C>T	Intron 2	–	0.01
i	c.512G>A	3	Arg171Gln	0.01
j	c.768T>C	4	Leu256Leu	0.01
k	c.824+31T>C	Intron 5	–	n/a
l	c.913−3C>G	Intron 6	–	0.02
m	c.1026G>A	7	Ala342Ala	0.01
n	c.1050−92C>T	Intron 7	–	0.03
o	c.1050−3C>G	Intron 7	–	0.02
p	c.1101A>C	8	Val367Val	n/a
q	c.1254C>T	9	Asp418Asp	0.42
r	c.1296G>A	9	Leu432Leu	0.01
s	c.1299T>C	9	His433His	0.01
t	c.1350+103G>C	Intron 9	–	0.42
u	c.1434C>T	10	Gly478Gly	n/a
v	c.1621G>A	10	Ala541Thr	0.04
w	c.1764G>A	10	Lys588Lys	n/a
x	c.1833*305_1833*307delCTC	10	–	0.05

n/a – Data not available. (From Lemos M and Thakker RV: Multiple Endocrine Neoplasia Type 1 (MEN1): Analysis of 1336 Mutations Reported in the First Decade Following Identification of the Gene. *Hum Mutat* 29: 22–32, 2008, with permission). (Lemos and Thakker, 2008).

a Polymorphism letter as referred to in Fig. 3.

b Polymorphisms are numbered in relation to the *MEN1* cDNA reference sequence (GenBank accession number NM_130799.1), whereby nucleotide +1 corresponds to the A of the ATG-translation initiation codon.

c Frequency presented in first literature report.

**Table 5:** Summary of biochemical and radiological screening guidelines in individuals at high risk of developing MEN1.

Tumor	Age to begin (yr)	Biochemical test (annually)	Imaging test (every 3 years)
Parathyroid Pancreatic neuroendocrine	8	Calcium, PTH	None
Gastrinoma	20	Gastrin (±gastric acid output)	None
Insulinoma	5	Fasting glucose, insulin	None
Other enteropancreatic	<10	Chromogranin-A; pancreatic polypeptide, glucagon; VIP	MRI, CT or EUS (annually)
Anterior pituitary	5	Prolactin, IGF-1	MRI (every 3 years)
Adrenal	<10	None, unless symptoms or signs of functioning tumor and/or tumor >1 cm are identified	MRI or CT annually with pancreatic imaging
Foregut carcinoid	20	None	CT or MRI (every 1–2 years)

CT, computer tomography; MRI, magnetic resonance imaging; EUS, endoscopic ultrasound. Reproduced from Thakker RV: Clinical Practice Guidelines for Multiple Endocrine Neoplasia Type 1 (MEN1). *J Clin Endocrinol Metab* 97: 2990–3011, ©2012. The Endocrine Society (Thakker et al., 2012).

**Table 6:** Functions of menin indicated by direct interactions with proteins and other molecules.

Function^a^	Interacting partner
Transcription regulation	JunD
	NF-κB (P50, P52, P65)
	pem
	SIN3A
	HDAC
	Smad1
	Smad3
	Smad5
	Runx2
	MLL histone methyl-transferase complex
	ER-Alpha
	CHES1
	Double-stranded DNA

Genome stability	RPA2
	FANCD2

Cell division	NMMHC II-A
	GFAP
	Vimentin

Cell cycle control	NM23 (A)
	ASK

Epigenetic regulation	MLL histone methyl-transferase complex ER-Alpha HDAC

a Functions reported include involvement in cell cycle withdrawal, decrease of cell motility, cell differentiation, apoptosis and DNA repair. (Adapted from Lemos M and Thakker RV: Multiple Endocrine Neoplasia Type 1 (MEN1): Analysis of 1336 Mutations Reported in the First Decade Following Identification of the Gene. Human Mutation 29: 22–32, 2008, with permission) (Lemos and Thakker, 2008).
